# Common dysregulation of Wnt/Frizzled receptor elements in human hepatocellular carcinoma

**DOI:** 10.1038/sj.bjc.6604422

**Published:** 2008-06-24

**Authors:** A Bengochea, M M de Souza, L Lefrançois, E Le Roux, O Galy, I Chemin, M Kim, J R Wands, C Trepo, P Hainaut, J-Y Scoazec, L Vitvitski, P Merle

**Affiliations:** 1 INSERM, U871, Molecular Physiopathology and New Therapies in Viral Hepatitis, 151 cours Albert Thomas, Lyon, F-69424, France; 2 Université Claude Bernard Lyon-1, Faculté de Médecine Laennec, IFR62, Lyon, F-69008, France; 3 IARC, Molecular Carcinogenesis, Lyon, France; 4 Department of Medicine, The Liver Research Center, Brown Medical School, Providence, Rhode Island, USA; 5 Hepatology Unit, Hospices Civils de Lyon, Hotel-Dieu Hospital, Lyon, F-69002, France; 6 Anatomopathology Laboratory, Edouard Hérriot Hospital, Lyon, France

**Keywords:** hepatocellular carcinoma, WNT, Frizzled

## Abstract

Dysregulation of growth factors and their receptors is central to human hepatocellular carcinoma (HCC). We previously demonstrated that the Frizzled-7 membrane receptor mediating the Wnt signalling can activate the *β*-catenin pathway and promotes malignancy in human hepatitis B virus-related HCCs. Expression patterns of all the 10 Frizzled receptors, and their extracellular soluble autoparacrine regulators (19 Wnt activators and 4 sFRP inhibitors) were assessed by real-time RT–PCR in 62 human HCC of different etiologies and their matched peritumorous areas. Immunostaining was performed to localise Frizzled on cell types in liver tissues. Regulation of three known Frizzled-dependent pathways (*β*-catenin, protein kinase C, and C-Jun NH_2_-terminal kinase) was measured in tissues by western blot. We found that eight Frizzled-potentially activating events were pleiotropically dysregulated in 95% HCC and 68% peritumours as compared to normal livers (upregulations of *Frizzled-3/6/7* and *Wnt3/4/5a*, or downregulation of *sFRP1/5*), accumulating gradually with severity of fibrosis in peritumours and loss of differentiation status in tumours. The hepatocytes supported the Wnt/Frizzled signalling since specifically overexpressing Frizzled receptors in liver tissues. Dysregulation of the eight Frizzled-potentially activating events was associated with differential activation of the three known Frizzled-dependent pathways. This study provides an extensive analysis of the Wnt/Frizzled receptor elements and reveals that the dysregulation may be one of the most common and earliest events described thus far during hepatocarcinogenesis.

Hepatocellular carcinoma (HCC) is one of the most frequent tumours worldwide. It develops mostly in cirrhotic livers, whose risk factors include chronic infection by hepatitis B and C viruses (HBV and HCV), and nonviral liver diseases (Llovet *et al*, 2003; Bosch *et al*, 2005). Unfortunately, cellular mechanisms of hepatocarcinogenesis remain poorly understood. Initially, a variety of genetic and epigenetic alterations were detected in HCCs and to a lesser extent in preneoplastic cirrhotic livers. Later on, DNA microarray analysis led to an extensive integrative approach, leading to the identification of clusters of HCCs associated with patterns of gene expression that allow comparison between HCC phenotypes in experimental and human HCCs (Lee and Thorgeirsson, 2006). In parallel, recent progress has been achieved in understanding the essential role and function that represents dysregulation of pleiotropic growth factors – that is, the insulin-like growth factor-II (IGF-II), hepatocyte growth factor (HGF), transforming growth factor-*α* (TGF-*α*), transforming growth factor-*β* (TGF-*β*), and wingless (Wnt) – in contributing to proliferation and antiapoptotic behaviour of HCC cells (Breuhahn *et al*, 2006).

Concerning the Wnt signalling cascade, the binding of one among the 19 known extracellular soluble secreted Wnt ligands (WNT) to one or more among the 10 Frizzled receptors (FZD) in cooperation or not with LRP coreceptors (low-density lipoprotein receptor (LDLR)-related protein), LRP-5 or LRP-6, can differentially lead to the activation of either the canonical *β*-catenin, or the noncanonical c-Jun N-terminal kinase (JNK) and protein kinase C (PKC) pathways, which may control the tumour phenotype. In contrast, four sFRP (secreted Frizzled-related proteins), which are extracellular soluble factors, can bind WNT and thereby downregulate their ability to activate FZD (Jones and Jomary, 2002). Different studies have clearly shown the upregulation of WNT/FZD elements in different types of cancers, highlighting their direct role in carcinogenesis as witnessed by the activation of oncogenic pathways and control of the cancerous phenotype (Tanaka *et al*, 1998; Kirikoshi *et al*, 2001; Holcombe *et al*, 2002; Weeraratna *et al*, 2002; Qiang *et al*, 2003; Lu *et al*, 2004; Zeng ZY *et al*, 2007). However, the expression patterns of all the WNT/FZD family members have not been extensively evaluated in human HCC so far.

We have previously shown the activation of *β*-catenin by overexpression of *FZD7* in human HBV-related HCCs as well as in several transgenic mouse models of hepatocarcinogenesis (Merle *et al*, 2004; Merle *et al*, 2005). Additionally, we have shown that FZD7 can be activated *in vitro* by binding of the WNT3 ligand (Kim *et al*, 2008). In this study, the expression patterns of all 19 *WNT*, 10 *FZD*, two *LRP,* and four *sFRP* genes have been determined in HCCs and in their matched surrounding precancerous peritumorous areas, by comparison to normal liver (NL) tissues. This revealed that three different FZD (FZD3, 6, and 7) are commonly upregulated in HCC cells, in association with the activation of potentially FZD-dependent cascades such as the canonical *β*-catenin, as well as the noncanonical PKC and JNK pathways. Furthermore, these three receptors were overexpressed concomitantly with either upregulation of three Wnt agonists (WNT3, 4, and 5A) or downregulation of two Wnt antagonists (sFRP1 and 5). We propose that accumulation of these events is independent of aetiologic factors, correlates more closely with progression from cirrhosis to HCC and ultimately directly relates to the loss of differentiation of such tumours. In parallel, we have shown that the WNT/FZD/sFRP expression patterns did not correlate to the *TP53* and *β-catenin* gene mutation status in the HCC samples, these genes being the most frequently altered tumour suppressor and proto-oncogene in HCC (Laurent-Puig and Zucman-Rossi, 2006).

## Materials and methods

### Human liver tissues

Sixty two frozen HCCs surgically resected from different individuals were obtained from Thailand (International Agency for Research on Cancer) (*n=*10) and France (National Resource Biological Center) (*n=*52), a written consent being obtained before surgery. Tissue samples were obtained and characterized as tumorous (T) or matched peritumorous liver parenchymas (pT). NL came from parenchyma surrounding surgically resected focal nodular hyperplasia from different individuals (*n=*9). HCCs were due to HBV (*n=*18) or HCV (*n=*20) infection as defined by positivity for HBs antigen or anti-HCV antibodies in serum, respectively, whereas others were presumed as nonviral-related (NBNC) tumours (*n=*24).

### Histological analysis

Histological analysis was performed on T to confirm the diagnosis of HCC and to classify them as to their differentiation status. Furthermore, histology served on pT as a method of assessing fibrosis stage using the Metavir criteria (The French METAVIR Cooperative Study Group, 1994), and to further ensure the absence of microscopic tumour invasion.

### Cell lines

The human HCC cell lines Huh7, Focus, PLC/PRF/5, Hep3B, as well as the hepatoblastoma HepG2 cell line, were grown in DMEM F-12, 1X-MEM nonessential amino-acid solution, 200 mM L-glutamine, 1 × -sodium-pyruvate, 1% (vol/vol) penicillin/streptomycin (Invitrogen), and supplemented with 10% (vol/vol) fetal calf serum (Sigma). Human primary hepatocytes were cultured in HCM Bullet-kit hepatocytes culture medium (Cambrex).

### Semi-quantitative real-time RT–PCR

We assessed the expression of the 19 *WNT*, 4 *sFRP*, 10 *FZD* and 2 *LRP* genes in cell lines, and liver tissues (62 T and 62 pT) as determined by an arbitrary value (AV) Additionally, a cohort of nine NL served as controls which gave cutoff values equal to mean±2 s.d. (*α*=0.05) for upregulation or downregulation of the different genes in pT and T. Total RNA extractions as well as cDNA synthesis were prepared as described previously (Merle *et al*, 2004). PCR reactions were performed by 35 cycles (95°C 15 s, 60°C 1 min) using the MyIQ™ Real Time PCR Detection System (Bio-Rad) with a mix composed of 1X-Quantitech Sybr Green PCR Kit (Qiagen), primers at either 500 nM (*Wnt*, *sFRP*, *FZD,* and *LRP*) or 800 nM (*18S RNA*), and 12.5 ng cDNA (equivalent total RNA) from unknown samples. Each PCR run included the unknown cDNAs and a nontemplate control (RNA sample treated with DNase without RT step) to check for the absence of genomic DNA contamination. Regarding the specificity of reactions, melt curves were analysed for each sample and the corresponding PCR products of the positive controls were cloned into the pCR®2.1 Vector (Invitrogen, Life Technology) and sequenced. Primers for *sFRP* genes were selected using the previously described strategy (Merle *et al*, 2004). Primers for *FZD7* and *18SrRNA* were previously published (Merle *et al*, 2004), as well as those for *Wnt*, *FZD* (except *FZD7*), and *LRP* (Lu *et al*, 2004) with slight modifications to obtain identical annealing temperature and PCR amplification efficiency as for *FZD7*, *18S rRNA,* and *sFRP* primers (Supplementary online data no. 1). The amount of specific mRNA was quantified in unknown samples by using the comparative Ct method: the *Δ*Ct values from each tissue were obtained by subtracting the values for 18S Ct from the Ct of each tested gene. One difference in *Δ*Ct represents a two-fold difference in the level of mRNA as described in (Lu *et al*, 2004), this method is giving an AV for the expression of each gene per tissue.

### Immunostaining

Immunohistochemistry was used to localise FZD3/6/7 in T and pT tissues fixed in 10% neutral-buffered formalin and embedded in paraffin. After antigen unmasking by heating slices in a microwave oven in citrate buffer at pH 6.0 for 10 min, the 5 *μ*m sections were incubated overnight at 4°C with anti-FZD3 (1/150, LifeSpan, Biosciences, LS-A4454), anti-FZD6 (1/70, LifeSpan, Biosciences, LS-A4481) or anti-FZD7 (1/200, Sigma, F3679) rabbit polyclonal antibodies. Endogenous peroxidase activity was blocked with the Peroxidase-Blocking reagent (DakoCytomation) for 10 min and nonspecific antibody binding was blocked with 10% skimmed milk for 30 min at 20°C. The Antibody-Diluent reagent (DakoCytomation) served as a negative control under equivalent conditions in place of the primary antibody. A goat antirabbit IgG conjugated to peroxidase-labelled polymers diluted in Tris-HCl buffer (EnVision+ Dual Link System Peroxidase – DakoCytomation) was added according to the manufacturer's instructions. For amplification of the reaction, a soluble antigen–antibody of horseradish peroxidase antiperoxidase developed in the rabbit (Sigma) was used at 1/200 dilution for 30 min at 20°C. Colorimetric reaction was developed with 3,3′-diaminobenzidine (Liquid DAB+ Substrate Chromogen System- DakoCytomation) for 2 min. Slides were counterstained with Harris hematoxylin, dehydrated, and coverslipped with EUKITT (O Kindler GmbH & CO, Freiburg).

### Protein extraction and western blotting

We analysed by western-blotting the activaty status of the three FZD-dependent intracellular pathways – that is, *β*-catenin, PKC, JNK – in T showing overexpressing of FZD3, 6, and/or 7 by comparison with their matched pT. Samples were treated with 200 *μ*l of extraction buffer (30 mM Tris-pH 7.5, 150 mM NaCl, 1% NP-40, 0.5% Na deoxycholate, 0.1% SDS, 10% glycerol and 2 mM EDTA) containing protease inhibitors and phosphatase inhibitors (Roche Diagnostics). Protein concentration was determined with the BCA Protein Assay Kit (Pierce) using BSA as standard. Subcellular fractionations were performed as described previously (Maloney *et al*, 1998), and protein concentration was measured by the method of Bradford with the Bio-Rad Protein Assay Kit (Bio-Rad Laboratories, Hercules, USA) using BSA as standard. Aliquot of proteins were resolved on SDS–PAGE and transferred onto PVDF membranes (Amersham) by electroblotting. The membranes were blocked with 5% nonfat dry milk in Tris-buffered saline containing 0.1% Tween 20, and then probed with an antibody targeting total *β*-catenin (Transduction Laboratory) or phospho-Thr41/Ser45 *β*-catenin (Cell Signalling), pan-PKC (Santa Cruz Technology) or pan-phospho-PKC (Cell Signalling), JNK (R&D Systems) or phospho-JNK (R&D Systems). The corresponding horseradish peroxidase antibodies were incubated and revealed with the chemiluminescence imaging ECL (Sigma). All of the blots were standardized for equal protein loading by Red Ponceau staining and western blotting for *β*-actin (Sigma).

### PCR/DHPLC Sequencing of *CTNNB1* and *TP53* Genes

For denaturing high performance liquid chromatography (DHPLC) analysis, crude amplification products were denaturated by heating at 95°C then cooled to 25°C over 1 h. DHPLC analysis were performed by injecting 5–8 *μ*l of PCR products into a preheated reverse-phase column (DNASep Column, Transgenomic) equilibrated with an ion pairing agent TEAA 0.1 M (Triethylammonium acetate). DNA was removed from the column by a linear gradient of eluting buffer containing 25% acetonitrile at a constant flow rate of 0.9 ml min^−1^ and with 2% per min gradient increase. Transgenomic UV-detector identified the eluted DNA at 260 nm. All amplimers with different profile shapes or retention times were sequenced to confirm the putative sequence variations. Concerning PCR sequencing, primer pairs were designed for amplification of exons-4 to -9 for *TP53*, and exon-3 for *β-catenin* genes (Supplementary online data no. 2). Each PCR mix contained genomic DNA, 1.5 mM MgCl_2_, 0.2 mM each dNTP, 0.4 *μ*M each primer, 1X-PCR Buffer (Invitrogen) and 2U Platinum® Taq DNA Polymerase (Invitrogen) for a final reaction volume of 50 *μ*l. The PCR amplification for *TP53* exons was performed using the following conditions: initial denaturation at 94°C 2 min, 20 cycles (94°C 45 s, 63°C 30 s, and gradual decrease of 0.5°C per three cycles, 72°C 45 s) followed by 30 cycles (94°C 45 s, 60°C 30 s, and 72°C 45 s) and ending with an extension at 72°C 10 min. The cycling profile for *β-catenin* exon-3 amplification was the following: initial denaturation at 95°C 2 min, 20 cycles (95°C 30 s, 56°C 30 s, and gradual decrease of 0.4°C per two cycles, 72°C 30 s) followed by 30 cycles (95°C 30 s, 52°C 30 s, and 72°C 30 s) and ending with extension at 72°C 7 min.

### Statistical analysis

The Mann–Whitney *U*-test was used with StatView software Version 5.0 (SAS Institute Inc.), and *χ*^2^test when necessary. Tests were considered significant when their *P* values were <0.05.

## Results

### Expression patterns of *FZD*, *LRP*, *WNT,* and *sFRP* genes at the mRNA level in liver tissues by quantitative real-time RT–PCR

**Figure 1:**
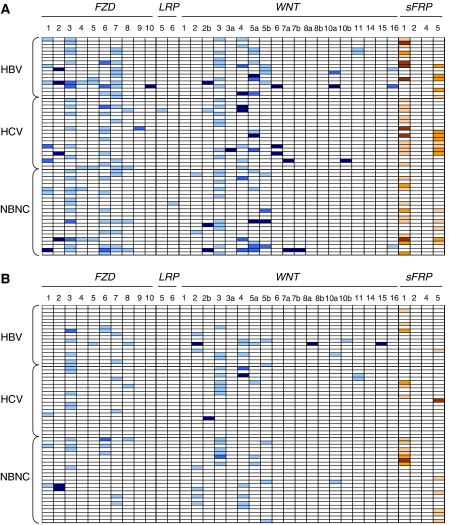
Expression patterns of *FZD*/*LRP*/*WNT*/*sFRP* genes in HCC tissues in T (**A**) and pT (**B**), by comparison to cutoff values obtained from NL. Each line represents a different HCC tissue depending on the aetiologic factor (lines 1–18 for HBV, lines 19–38 for HCV, and lines 39–42 for NBNC). Upregulation of genes are indicated in coloured boxes as light blue (one- to five-fold), medium dark blue (five- to 10-fold), or dark blue (>10-fold). Downregulation of genes are indicated in pink (five-fold), orange (five- to 10-fold), or brown (<10-fold).

Three different *FZD* genes were found frequently upregulated in T and pT by comparison to NL (>cut-off; Figure 1): *FZD3* (41% T, 23% pT), *FZD6* (31% T, 8% pT), and *FZD7* (33% T, 10% pT). By contrast, almost none of the samples showed any significant upregulation or downregulation of *LRP* genes in pT or T tissues by comparison to NL (< or >cut-off). Concerning the soluble extracellular regulators of the Frizzled membrane receptors, *WNT3, WNT4,* and *WNT5A*, potent activators, were strikingly found upregulated by comparison to NL (>cutoff value): *WNT3* (39% T, 25% pT), *WNT4* (20% T, 16% pT), and *WNT5A* (25% T, 7% pT). Among the potential inhibitors of the Frizzled receptors, two *sFRP* genes were found downregulated: *sFRP1* (53% T, 21% pT), and *sFRP5* (28% T, 12% pT).

**Figure 2:**
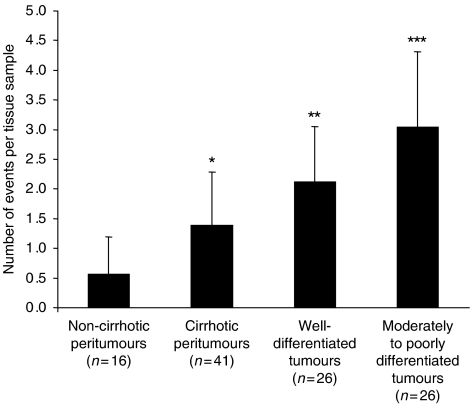
Evidence of accumulating WNT/FZD events with occurrence of cirrhosis in peritumorous tissues, with development of tumours and subsequently with severity of tumours in terms of dedifferentiation status. Bars (±s.d.) represent the average number of events – that is, upregulation of *FZD3*, *FZD6*, *FZD7*, *WNT3*, *WNT4*, and *WNT5A* or downregulation of *sFRP1*, *sFRP5* – per tissue sample. Comparisons were statistically assessed by the Mann–Whitney *U*-test : ^*^P<0.01 between noncirrhotic and cirrhotic peritumours; ^**^P<0.05 between cirrhotic peritumours and well-differentiated tumours; ^***^P<0.05 between well-differentiated and moderately/poorly differentiated tumours.

Taken together, these results demonstrated that when pooling the eight following events – that is, upregulation of potential activators (*FZD3, FZD6, FZD7, WNT3, WNT4,* and *WNT5A*) or repression of inhibitors (*sFRP1* and *sFRP5*) of the WNT/FZD signalling – one of them at least occurred in 68% pT and 95% T. Strikingly, each of these events accumulated with progression from noncirrhotic to cirrhotic tissues of peritumour areas (0.6±0.6 events per noncirrhotic pT *vs* 1.4±0.9 events per cirrhotic pT; *P*<0.01). They additionally carried on their accumulation within tumours (1.4±0.9 events per cirrhotic pT *vs* 2.1±0.9 per well-differentiated HCC, *P*<0.05), and subsequently with the loss of differentiation status of tumours (2.1 ±0.9 per well-differentiated HCC *vs* 3.0±1.3 events per moderately to poorly differentiated tumour; *P*<0.05) (Figure 2).

It is of note that, we carefully evaluated by serial tissue sections that none of the pT with elevated *WNT/FZD* or decreased *sFRP* expression levels had microscopic HCC tumour foci. Concerning relationship to aetiologic factors of HCC, with the notable exception of *FZD7* which showed higher rate of upregulation in HBV*vs* non-HBV-related HCC (59 *vs* 23%, CHI-2 test, *P=*0.035), *WNT3/4/5A*, *FZD3/6,* and *sFRP1/5* were statistically equally dysregulated between HBV, HCV, and NBNC-related HCCs.

### Correlation of *WNT/FZD/sFRP* expression to mutation status of *β-Catenin* and *TP53* genes in HCCs

The *β-catenin* and *TP53* mutation status was determined in HCC and compared to *WNT/FZD/sFRP* expression patterns (Supplementary online data no. 3). The *β-catenin* gene was found mutated mainly in HCV-related HCC (HBV 17%, HCV 40%, and NBNC 21%), whereas TP53 mutations did not correlate with aetiologic factors (HBV 33%, HCV 30%, and NBNC 13%). There was no correlation between these mutations and a specific *WNT/FZD/sFRP* expression pattern in HCC.

### Cell specificity of Frizzled receptors and activity of Frizzled-dependent intracellular pathways

**Figure 3:**
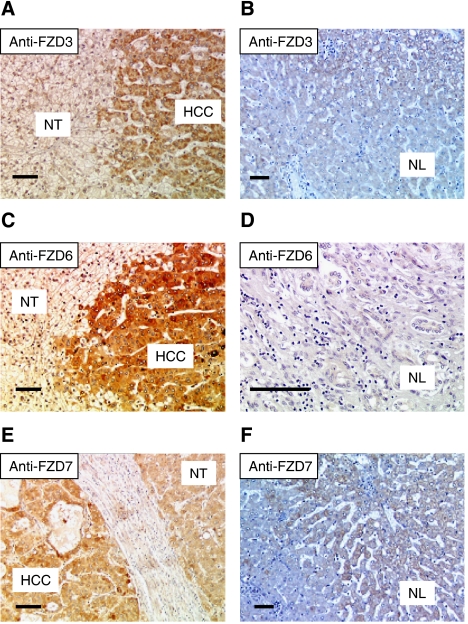
Representative examples of immunostaining for Frizzled receptors (FZD) in liver sections involving both T and pT: FZD3 (**A**), FZD6 (**C**) and FZD7 (**E**). Staining of NL is presented for comparison for (**B**, **D**, and **F**). FZD3 was detected mainly in HCC cells as compared to adjacent nontumorous hepatocytes (**A**). In the normal liver, only a faint labelling was observed in the perivenous area of the lobule (**B**). FZD6 was strongly expressed mainly in HCC cells as compared to adjacent nontumorous hepatocytes (**C**). In the normal liver, no expression was detected either in hepatocytes or normal biliary epithelial cells (**D**). FZD7 was highly expressed by HCC cells in a well-differentiated HCC (**E**). In the normal liver, faint labelling was observed in hepatocytes located in the perivenous region of the lobule (**F**). Size bars=500 *μ*m.

Immunostaining allowed at identifying the specific cells, which overexpressed FZD3, FZD6, and FZD7 proteins within T and pT tissues. As shown in Figure 3, these three receptors were highly expressed by HCC cells in T, and at a lesser extent by nontransformed hepatocytes in pT, whereas they were barely or not expressed by both nonhepatocytic cells and normal hepatocytes in NL. As a confirmatory result, quantitative real-time RT–PCR data showed the capability of cancerous HCC cell lines to express high steady state levels of FZD3, FZD6, and/or FZD7 mRNAs, whereas normal primary hepatocytes did not (Table 1).

**Figure 4:**
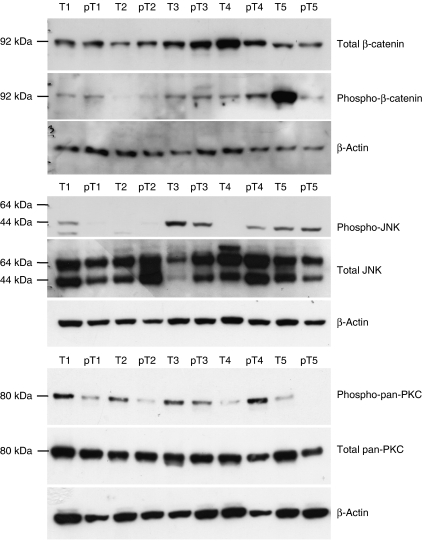
Western blot analysis of FZD-dependent intracellular pathways (*β*-catenin, PKC, and JNK). Activity was assessed in a panel of HCC (T) and their matched nontumorous liver parenchyma (pT). The *β*-catenin activity was assessed as the ratio between total-*β*-catenin and phsopho-*β*-catenin, whereas the PKC and JNK activities were assessed as the ratio between the phospho-PKC or phosphor-JNK and the total PKC or total JNK. All of the blots were standardized for equal protein loading by *β*-actin.

Next, we assessed the activation of the downstream pathways potentially controlled by the WNT/FZD signalling – that is, *β*-catenin, PKC and JNK – in liver tissues. To this aim, we examined a cohort of 15 paired samples (T + pT) where the previously identified WNT3/4/5A, FZD3/6/7, sFRP1/5 events gradually accumulated from pT towards the matched T, each pT serving as a matched negative control for the corresponding T. As expected, most of the tested T (13/15, 87%) showed higher activity of either *β*-catenin, PKC or JNK pathways than their matched pT (Table 2), as shown respectively by unphosphorylation of the cytosolic form of *β*-catenin at threonine-41/serine-45 residues, phosphorylation of PKC at serine-660 residue, and phosphorylation of JNK at threonine-183/tyrosine-185 residues (Figure 4). Before assessing the potential impact of the WNT/FZD signalling on the activity of *β*-catenin, we carefully checked that all tested T samples did not contain specific mutations within the serine/threonine residues of the *β-catenin* gene. In contrast, 3/4 (75%) T devoided of accumulation of WNT/FZD events did not show increased activity of one of the three pathways in T *vs* pT (Table 2).

## Discussion

We have demonstrated for the first time that dysregulation of the WNT/FZD receptor elements is an overall common feature in human hepatocarcinogenesis. This study has provided a comprehensive transcriptomic analysis of the FZD/LRP receptor complexes, and some of their potential autoparacrine regulators – that is, WNT and sFRP elements in human HCC. We have identified eight potential FZD activating events, each occurring in a significant proportion of HCCs (⩾20%) – that is, upregulation of *FZD3/6/7* and *WNT3/4/5A*, and repression of *sFRP1/sFRP5* – which accumulate with severity of the liver disease and tumour stage. Furthermore, we have shown that the FZD3/6/7-mediated signalling is supported by HCC cells within tumour liver tissues. Finally, we have observed that at least one over the three known different intracellular pathways potentially controlled by the WNT/FZD signalling during embryogenesis or carcinogenesis – that is, canonical *β*-catenin, or noncanonical PKC and JNK – appeared activated in a majority of the tested HCC tissues containing the accumulation of WNT/FZD element dysregulations.

Research on dysregulation of growth factors and their receptors aims at establishing the role and function of the corresponding signalling cascades in human hepatocarcinogenesis towards the identification of new therapeutic targets. In this view, we previously demonstrated the activation of the WNT/FZD signalling through *FZD7* overexpression in transgenic mouse models of HCC as well as in human HBV-related HCC tissues from Taiwan and South Africa (Merle *et al*, 2004, 2005). However, it remained questionable whether *FZD7*, among the ten known human *FZD* receptors, is the unique *FZD* involved in human hepatocarcinogenesis, and what happened in non-HBV-related HCCs. In this study, we have confirmed in tissues from Thailand and Western Europe that *FZD7* is overexpressed in a majority of HBV-related HCCs and, although at a lesser extent, in a significant proportion of HCV and nonvirus-related HCCs. At present, no information is available as to the possible interactions between HBV and the regulatory elements of *FZD7*, thus emphasising the need for additional investigation. Furthermore, we have shown that not only *FZD7* but also *FZD3* and *FZD6* are commonly overexpressed in HCCs. Noticeably, *FZD3,* and *FZD7* were found more closely related to the poorly differentiation status of tumours, indicating their possible involvement in the latter steps of hepatocarcinogenesis, and their possible role in the metastasis processes and epithelial-mesenchymal transition as recently reported for *FZD7* in colon tumours (Vincan *et al*, 2005). The potent soluble activators of *FZD3/6/7* are very likely those ligands to be found overexpressed in HCCs (*WNT3/4/5A*).

These data correlate with *FZD3/6* and *WNT3* found overexpressed in other malignancies as leukaemia, *FZD7* in solid tumours like nasopharynx, oesophagus, and stomach carcinomas, *WNT5A* in leukaemia, melanoma, and nasopharynx carcinoma (Tanaka *et al*, 1998; Kirikoshi *et al*, 2001; Holcombe *et al*, 2002; Weeraratna *et al*, 2002; Qiang *et al*, 2003; Lu *et al*, 2004; Zeng ZY *et al*, 2007), whereas *WNT4* has been previously found overexpressed in pancreatic cancers (Pasca di Magliano *et al*, 2007). Concerning the soluble activators of *FZD*, previous studies have shown the common downregulation of *sFRP1* due to promoter hypermethylation in HCC as well as in colon cancer (Suzuki *et al*, 2004; Shih *et al*, 2006, 2007). In this study, we have found that although *sFRP1* was commonly downregulated in pT and T, another inhibitor such as *sFRP5* shared the same expression pattern of repression. Finally, as the *WNT/FZD/sFRP* signalling elements are dysregulated in most HCCs, we have been unable to find a specific association between their expression pattern and the occurrence of either *TP53* or *β-catenin* gene mutations, which paradoxically had been shown in previous studies as dichotomizing HCC tumours in different clusters (Laurent-Puig *et al*, 2001).

Of interest was the progressive accumulation of the FZD activating events (*WNT3/4/5A* and *FZD3/6/7* upregulations and *sFRP1/5* repression) with severity of the liver disease and the tumour stage. These results suggest that the FZD activating event accumulation may contribute to enhance the activity of the downstream pathways that may control the cancerous phenotype. Thus, we tried to assess the differential activity of the various potentially FZD-dependent intracellular pathways (*β*-catenin, PKC, and JNK) between HCC tumours (T) and their matched nontumorous tissues (pT). The canonical WNT signals are transduced through FZD family receptors and LRP5/LRP6 coreceptor to the *β*-catenin signalling cascade. In the absence of canonical WNT signalling, *β*–catenin combined to APC and AXIN is phosphorylated by casein kinase Ia (CKIa) and glycogen synthase kinase 3*β* (GSK3 *β*), and polyubiquitinated by hTRCP1 or hTRCP2 for degradation by the proteasome. In the presence of canonical WNT signalling, Dishevelled (DVL) is phosphorylated by CKIa and complexes with FZD. Subsequently, *β*-catenin is released from phosphorylation by CKIa and GSK3*β* for stabilisation, cytosolic accumulation and nuclear translocation. Nuclear *β*-catenin is complexed with T-cell factor/lymphoid enhancer factor (TCF/LEF) family transcription factors and other proteins (BCL9 and BCL9L Legless family docking proteins, and the PYGO1 and PYGO2 PYGO-family coactivators). The TCF/LEF-*β*-catenin-Legless-PYGO nuclear complex is the effector of the canonical WNT signalling pathway to activate the transcription of target genes such as FGF20, DKK1, WISP1, MYC, and CCND1. Noncanonical WNT signals are transduced through FZD family receptors and coreceptors, such as ROR2 and RYK. Small G proteins (RHOA, RHOU, RAC, and CDC42) and c-jun NH2-terminal kinase are the DVL-dependent effector molecules of the noncanonical pathway, whereas nemo-like kinase (NLK) and nuclear factor of activated T cells (NFAT) are the Ca2+-dependent effector molecules of noncanonical pathway. Small G proteins are implicated in the cytoskeletal reorganisation during invasion and metastasis. NLK phosphorylates TCF/LEF family transcription factors to inhibit the canonical WNT signalling pathway. NFAT transcription factor is implicated in the convergent extension during early embryogenesis as well as in the metastasis during carcinogenesis. Noncanonical WNT signalling pathway, transduced to a variety of DVL- or Ca2+-dependent cascades, is overlapping with the planar cell polarity signalling pathway (Katoh and Katoh, 2007a). It remains questionable the accuracy of the role of these three FZD potentially dependent pathways in human HCC. Evidence is accumulating to the fact that alterations of the Wnt/*β*-catenin pathway, due or unrelated to *β*-catenin gene mutation, is a common event in hepatocarcinogenesis and is associated with the clinical and pathological features of the disease (Hsu *et al*, 2000; Devereux *et al*, 2001; Laurent-Puig *et al*, 2001; Wong *et al*, 2001; Inagawa *et al*, 2002). As to the PKC and JNK pathways, their role and prevalence of activation remain poorly known in human hepatocarcinogenesis, although some studies have underlined their potentially oncogenic properties (Delpuech *et al*, 2002; Guo *et al*, 2005; Wu *et al*, 2008). In this study, we did observe an increment in activity of one or more of the three WNT/FZD potentially dependent pathways (*β*-catenin, PKC, and JNK) in a majority of HCCs with the accumulation of dysregulations of WNT/FZD elements and the absence of *β-catenin* gene mutation, by comparison to their matched pT. These data suggest that the WNT/FZD-mediating signalling may differentially activate these pathways, although other mechanisms independent of the WNT/FZD signalling may interfere.

Whereas certain WNTs appear to play predominant roles in initiating canonical (*β*-catenin) *vs* noncanonical (PKC and JNK) pathways, the relative extents to which most WNTs or FZDs participate in one and/or the other pathways has not been well established. Many of FZD receptors, including FZD3 and FZD7, are able to activate the canonical *β*-catenin pathway, whereas data are not so obvious for FZD6. The noncanonical PKC and JNK pathways have been clearly shown as potentially activated by some FZD family members as FZD7, whereas it remains ambiguous for FZD3 and FZD6 (He *et al*, 1998; Winklbauer *et al*, 2001; Kinoshita *et al*, 2003; Merle *et al*, 2004; Katoh, 2007; Katoh and Katoh, 2007b). We have previously reported that the FZD7-mediated signalling was able to control the cancerous phenotype of human HCC cell lines through *β*-catenin protein regulation, and that one of the Wnt ligand candidate activators is WNT3 (Merle *et al*, 2004, 2005; Kim *et al*, 2008). Nothing is clear about the functional interaction between FZD7 and WNT4 or WNT5A. Others have observed that the WNT3/FZD3 combination could activate the canonical *β*-catenin pathway in malignant lymphocytes (Lu *et al*, 2004). Although WNT5A was shown as a natural ligand of FZD3 that triggers the PI3K/Akt signal (Kawasaki *et al*, 2007), nothing is evident about the WNT4/FZD3 interactions. Finally, there is probably a very complex network of interplays between Wnt-signalling elements and the cellular machinery. Wnt signals are context-dependently transduced to the canonical and noncanonical pathways based on the expression profile of *WNT*, *SFRP*, and *FZD* genes for instance, and the activity of cytoplasmic WNT signal regulators. Additionally, a higher level of complexity is brought on by the fact that switches of noncanonical signalling molecules may occur during embryogenesis and carcinogenesis (Katoh and Katoh, 2007b).

Importantly, our immunohistochemical approach showed a clear and almost exclusive FZD3/6/7-staining in HCC cells in tumours and hepatocytes in peritumours, demonstrating that the FZD3/6/7-mediated signalling could be specifically carried out by cancerous cells within HCC tissues, but not by mesenchymal or epithelial biliary cells. In contrast, little is known about the cells secreting the WNT3/4/5 and sFRP1/5 factors within liver tissues, and additional studies would be warranted to identify them as active and/or resting states of various cell types within the liver – that is, hepatocytes, biliary epithelial, sinusoidal endothelial, stellate, and Kupffer cells – as previously described in normal mouse livers (Zeng G *et al*, 2007).

Finally, we have described for the first time that different potential FZD activating events occurring in many human HCC tissues and associated with the activation of different FZD-dependent pathways, which may play a role in hepatocarcinogenesis. This does appear to be an early event that amplifies during liver disease and tumour progression, suggesting a key role in hepatocarcinogenesis. Nonetheless, additional experiments will be required to assess the impact of the different WNT3/4/5A and FZD3/6/7 combinations for activation of the FZD-dependent pathways and control of the cancerous phenotype in a specific context-dependent cell state (HCC cells, nontransformed progenitors, and primary cells). This will be a prerequisite before the designation of FZD receptors as exciting targets for therapeutic approaches of HCC.

## Figures and Tables

**Table 1:** Steady state levels of mRNAs for *FZD3*, *FZD6,* and *FZD7* genes as assessed by quantitative real-time RT–PCR in different human hepatoma cell lines in comparison to normal human primary hepatocytes

**Hepatocytes**	**FZD3**	**FZD6**	**FZD7**
Normal primary hepatocytes	+/−	+	+/−
Hepatoma HepG2	++	++++	+/−
Hepatoma Hep3B	+	+++++	+
Hepatoma PLC/PRF/5	++	+	++
Hepatoma Focus	++	++++	++
Hepatoma Huh7	++	+	+++

As described in Material and Methods, any gene *mRNA* steady state level was expressed as arbitrary value (AV). A semi-quantitative scale was proposed for gene expression: (−): AV=0; (+/−): AV=0 to 20; (+): AV=21 to 50; (++): AV=51 to 100; (+++): AV=101 to 150; (++++): AV=151 to 200; (+++++): AV > 200.

**Table 2:** Accumulation of Wnt/Frizzled-signallling dysregulation events (upregulation of *FZD*3, *FZD6*, *FZD7*, *WNT3*, *WNT4*, and *WNT5A* or repression of *sFRP1* and *sFRP5*) between HCC tumours (T) and their matched nontumorous counterparts (pT), and increased activity of different potentially Frizzled-dependent intracellular cascades in T in comparison to pT

**WNT3/4/5A, FZD3/6/7 and sFRP1/5 dysregulation events in T *vs* pT (# HCC ID)**	***β*-catenin mutation status in T**	**Increased activity of *β*-catenin in T *vs* pT**	**Increased activity of PKC in T *vs* pT**	**Increased activity of JNK in T *vs* pT**
*FZD3/6, sFRP1/5 vs sFRP5 (no. 50)*	−	+	−	+
*None vs none (no. 51)*	−	−	−	−
*sFRP1 vs WNT4 (no. 52)*	−	−	−	+
*WNT3 vs none (no. 54)*	−	−	−	−
*FZD3/6/7, WNT3/5A vs sFRP5 (no. 60)*	−	+	−	−
*FZD6, sFRP5 vs FZD3 (no. 30)*	−	−	+	−
*FZD7, WNT3, sFRP1 vs WNT3 (no. 25)*	−	−	−	+
*FZD7, WNT4 vs none (no. 36)*	−	+	+	+
*FZD3/6/7, WNT4/5A vs none (no. 61)*	−	−	−	−
*WNT3/4, sFRP1/5 vs FZD7 (no. 9)*	−	−	+	−
*FZD3/7, WNT4 vs none (no. 11)*	−	+	+	−
*FZD7, WNT5A, sFRP5 vs WNT4/5A (no. 12)*	−	−	+	+
*WNT3/5A, sFRP1/5 vs none (no. 13)*	−	+	−	−
*FZD6/7, WNT4/5A vs FZD3/7 (no. 17)*	−	+	+	−
*FZD3/7 vs none (no. 14)*	−	−	−	+
*FZD7, sFRP5 vs FZD3 (no. 18)*	−	+	−	+
*sFRP5 vs WNT3 (no. 16)*	−	−	−	−
*FZD7, WNT4 vs none (no. 20)*	−	+	−	−
*None vs none (no. 49)*	−	−	−	−

The *β*-catenin activity was assessed by western blot analysis as a ratio between the level of total *β*-catenin and phospho-*β*-catenin (Thr41/Ser45). The cytosolic fraction derived from HCC tissues (T and pT) was used for immunoblotting. The *β*-catenin gene status was wild type as revealed by the sequencing of exon-3. The PKC and JNK activities were assessed by western blot analysis as a ratio between the levels of pan-phospho-PKC (Ser660) or phospho-JNK (Thr183/tyr185), and pan-PKC or JNK. (+) Activity of each pathway was considered as higher in T *vs* pT when the western blot analysis value was increased by at least 25% in T *vs* pT. Note the increased of activity of one or more pathways in most of T *vs* pT.

## Supplementary Material

Supplementary online data no. 1

Supplementary online data no. 2

Supplementary online data no. 3
