# Supplementary material for: Common dysregulation of Wnt/Frizzled receptor elements in human hepatocellular carcinoma
Source: Br J Cancer. 2008 Jun 24;99(1):143–50. doi: 10.1038/sj.bjc.6604422 (PMC2453022; doi:10.1038/sj.bjc.6604422)
Supplement: Supplementary online data no. 1 [file 6604422x1.doc]

**Supplementary online data #1.** Primers used for semi-quantitative real-time PCR.

| Gene | Forward primer sequences  (5’→ 3’) | Reverse primer sequences  (5’→ 3’) |
| --- | --- | --- |
| *FZD1* | caccttgtgagccgaccaa | cagcactgaccaaatgccaat |
| *FZD2* | tttctgggcgagcgtgat | aaacgcgtctcctcctgtga |
| *FZD3* | tggctatggtggatgatcaaag | tggaggctgccgtggta |
| *FZD4* | ggcggcatgtgtctttcagt | gaatttgctgcagttcagactctct |
| *FZD5* | cgcgagcacaaccacatc | agaagtagaccaggaggaagacgat |
| *FZD6* | acaagctgaaggtcatttccaaa | gctactgcagaagtgccatgat |
| *FZD7* | caacggcctgatgtactttaagg | catgtccaccaggtaggtgaga |
| *FZD8* | gctcggtcatcaagcaacag | acggtgtagagcacggtgaac |
| *FZD9* | gcgctcaagaccatcgtcat | atccgtgctggccacgta |
| *FZD10* | gccgccatcagctccat | tcatgttgtagccgatgtcctt |
| *LRP5* | cgtgattgccgacgatctcc | tccggccgctagtcttgtc |
| *LRP6* | gttatgtgccacacccaagttct | ctgagggagctgatcattgattta |
| *WNT1* | cgaacctgcttacagactccaa | tcagacgccgctgtttgcg |
| *WNT2* | ggatgaccaagtgtgggtgtaag | gtgcacatccagagcttccag |
| *WNT2B* | ggcacgagtgatctgtgacaata | cgcatgatgtctgggtaacgc |
| *WNT3* | acttcggcgtgttagtgtcc | catttgaggtgcatgtggtc |
| *WNT3A* | gcccgtgctggacaaagct | ttctgcacatgagcgtgtcact |
| *WNT4* | ggaggagacgtgcgagaaac | ccaggttccgcttgcacatct |
| *WNT5A* | ttctccttcgcccaggttgtaa | cttctgacatctgaacagggtattc |
| *WNT5B* | ccaactcctggtggtcattagc | ctgggcaccgatgataaacatc |
| *WNT6* | cttccgccgctggaattgc | aggccgtctcccgaatgtc |
| *WNT7A* | cgacgccatcatcgtcatagga | ggccattgcggaactgaaactg |
| *WNT7B* | gtgaagctcggagcactgtca | aggccaggaatcttgttgcaga |
| *WNT8A* | cgcagaggcggaactgatctt | cgaccctctgtgccatagatg |
| *WNT8B* | aatcgggagacagcatttgtgca | atctccaaggctgcagtttctagt |
| *WNT10A* | ctgggtgctcctgttcttccta | gaggcggaggtccagaatg |
| *WNT10B* | cctcgcgggtctcctgttc | aggcccagaatctcattgcttag |
| *WNT11* | cgtgtgctatggcatcaagtgg | gcagtgttgcgtctggttcag |
| *WNT14* | gggcagacggtcaagcaag | cccagccttgatcaccttcaca |
| *WNT15* | gcctgcttgagtgccagttt | ctctcttgagcaggcccatc |
| *WNT16* | gccaatttgccgctgaacagc | cggcagcaggtacggtttc |
| *sFRP1* | aggcggatttccctggtagt | tagggcaaccacggactctt |
| *sFRP2* | atttctgctccgggatctca | tggagcagctaggagtgtgc |
| *sFRP4* | gctgcaatgaggtcacaacg | gttcgagggatgggtgatga |
| *sFRP5* | gtcaaaatgcgcatcaagga | ttctgggctccaatcagctt |
| *18SrRNA* | ggacacggacaggattgaca | acccacggaatcgagaaaga |
