# Supplementary material for: Common dysregulation of Wnt/Frizzled receptor elements in human hepatocellular carcinoma
Source: Br J Cancer. 2008 Jun 24;99(1):143–50. doi: 10.1038/sj.bjc.6604422 (PMC2453022; doi:10.1038/sj.bjc.6604422)
Supplement: Supplementary online data no. 2 [file 6604422x2.doc]

**Supplementary online data #2.** Primers and DHPLC conditions used for *TP53* and *β-catenin*.

| **Gene** | **Exons** | **Primer sequences**  **(5’→ 3’)** | **Amplicon size (bp)** | **DHPLC gradient (% elution buffer)** | **DHPLC temperatures (°C)** |
| --- | --- | --- | --- | --- | --- |
| *TP53* | Exon 4 | tgaggacctggtcctctgac (forward)  agaggaatcccaaagttcca (reverse) | 412 | 57, 52, 46 (start)  65, 60, 54 (stop) | 62, 65, 68 |
|  | Exon 5-6 | tgttcacttgtgccctgact (forward)  ttaacccctcctcccagaga (reverse) | 467 | 58, 52, 50 (start)  64, 60, 58 (stop) | 62, 66, 68 |
|  | Exon 7 | cttgccacaggtctccccaa (forward)  aggggtcagcggcaagcaga (reverse) | 237 | 53 (start)  61 (stop) | 64 |
|  | Exon 8-9 | ttgggagtagatggagcct (forward)  agtgttagactggaaacttt (reverse) | 445 | 56, 54, 49 (start)  64, 62, 57 (stop) | 60, 62, 68 |
| *β-catenin* | Exon 3 | ccaatctactaatgctaatactg (forward)  ctgcattctgactttcagtaagg (reverse) | 310 | 52, 49 (start)  60, 57 (stop) | 60, 63 |
