# Supplementary material for: Common dysregulation of Wnt/Frizzled receptor elements in human hepatocellular carcinoma
Source: Br J Cancer. 2008 Jun 24;99(1):143–50. doi: 10.1038/sj.bjc.6604422 (PMC2453022; doi:10.1038/sj.bjc.6604422)
Supplement: Supplementary online data no. 3 [file 6604422x3.doc]

**Supplementary online data #3.** Correlations between HCC (T and pT) and their respective : etiologic factors (HBV, HCV, NBNC), histopathology of T (G1, well; G2, moderate; G3, poor differentiation status) and pT (F1 to F4 fibrosis stage following Metavir criteria), *β-catenin* and *TP53* gene mutations (del = deletion), and the number of the following events per tissue (up-regulation of *FZD*3, *FZD6*, *FZD7*, *WNT3*, *WNT4*, *WNT5A*, or down-regulation of *sFRP1*, *sFRP5*). ND = not determined.

| HCC ID  T / pT | Etiology | T differentiation /  pT fibrosis stage | *β-catenin*  mutations | *TP53*  mutations | *Number of* WNT3/4/5A*,* FZD3/6/7 *and* sFRP1/5 *events in T/pT* |
| --- | --- | --- | --- | --- | --- |
| *#1* | HBV | G1 / F2 | - / - | - / - | 4 / 0 |
| *#2* | HBV | G2 / F4 | codon 41 / - | - / - | 2 / 1 |
| *#3* | HBV | G2 / F3 | - / - | - / - | 2 / 0 |
| *#4* | HBV | G3 / F2 | - / - | - / - | 3 / 0 |
| *#5* | HBV | G3 / F4 | - / - | codon 249 / - | 5 / 0 |
| *#6* | HBV | G1 / F1 | - / - | - / - | 1 / 1 |
| *#7* | HBV | ND / F4 | codon 33 / - | codon 278 / - | 1 / 2 |
| *#8* | HBV | G2 / ND | - / - | - / - | 6 / 3 |
| *#9* | HBV | G2 / F4 | - / - | - / - | 4 / 1 |
| *#10* | HBV | G3 / F4 | - / - | codon 246 / - | 1 / 0 |
| *#11* | HBV | G1 / F2 | - / - | codon 108 / - | 1 / 2 |
| *#12* | HBV | G2 / F4 | - / - | - / - | 3 / 2 |
| *#13* | HBV | G2 / F4 | - / - | - / - | 4 / 0 |
| *#14* | HBV | G2 / F1 | - / - | codon 294 / - | 2 / 1 |
| *#15* | HBV | G2 / F3 | codon 32 / - | - / - | 4 / 3 |
| *#16* | HBV | G2 / F4 | - / - | exons 5-6 del / - | 1 / 1 |
| *#17* | HBV | G2 / F2 | - / - | - / - | 4 / 2 |
| *#18* | HBV | G1 / F4 | - / - | - / - | 2 / 1 |
| *#19* | HCV | G1 / F4 | - / - | - / - | 3 / 3 |
| *#20* | HCV | G1 / F4 | - / - | codon 205 / - | 0 / 1 |
| *#21* | HCV | G1 / F4 | - / - | - / - | 6 / 2 |
| *#22* | HCV | G2 / F4 | - / - | - / - | 2 / 1 |
| *#23* | HCV | G1 / F4 | - / - | - / - | 3 / 3 |
| *#24* | HCV | G1 / F4 | - / - | - / - | 1 / 2 |
| *#25* | HCV | G2 / F4 | - / - | codon 115 / - | 3 / 1 |
| *#26* | HCV | G2 / F4 | codon 33 / - | - / - | 1 / 2 |
| *#27* | HCV | ND / F3 | - / - | codon 248 / - | 3 / 1 |
| *#28* | HCV | ND / F2 | codon 37 / - | codon 220 / - | 3 / 1 |
| *#29* | HCV | G2 / F4 | exon 3 del / - | - / - | 3 / 1 |
| *#30* | HCV | G1 / F2 | - / - | - / - | 2 / 1 |
| *#31* | HCV | G1 / F4 | codon 45 / - | - / - | 2 / 1 |
| *#32* | HCV | G2 / F1 | codon 32 / - | - / - | 4 / 0 |
| *#33* | HCV | G1 / F3 | codon 34 / - | - / - | 4 / 0 |
| *#34* | HCV | G1 / F3 | codon 34 / - | - / - | 3 / 0 |
| *#35* | HCV | G1 / F3 | - / - | codon 161 / - | 2 / 0 |
| *#36* | HCV | G1 / F4 | - / - | - / - | 2 / 0 |
| *#37* | HCV | G1 / F2 | codon 32 / - | - / - | 2 / 0 |
| *#38* | HCV | G1 / F4 | - / - | - / - | 2 / 0 |
| *#39* | NBNC | G2 / F4 | codon 33 / - | - / - | 4 / 4 |
| *#40* | NBNC | G1 / F4 | - / - | - / - | 2 / 2 |
| *#41* | NBNC | G1 / F4 | - / - | - / - | 4 / 2 |
| *#42* | NBNC | G2 / F4 | - / - | - / - | 1 / 3 |
| *#43* | NBNC | ND / ND | codon 45 / - | codon 33 / - | 1 / 3 |
| *#44* | NBNC | G2 / F4 | codon 33 / - | - / - | 4 / 3 |
| *#45* | NBNC | G2 / F4 | - / - | - / - | 2 / 1 |
| *#46* | NBNC | G1 / F4 | codon 45 / - | - / - | 1 / 3 |
| *#47* | NBNC | G1 / F4 | - / - | - / - | 1 / 1 |
| *#48* | NBNC | G1 / F4 | - / - | - / - | 2 / 0 |
| *#49* | NBNC | ND / F2 | - / - | - / - | 0 / 0 |
| *#50* | NBNC | G2 / F4 | - / - | - / - | 4 / 1 |
| *#51* | NBNC | G2 / F4 | - / - | codon 249 / - | 0 / 0 |
| *#52* | NBNC | G1 / F4 | - / - | - / - | 1 / 1 |
| *#53* | NBNC | G3 / F4 | exon 3 del / - | codon 280 / - | 7 / 0 |
| *#54* | NBNC | G1 / F4 | - / - | - / - | 1 / 0 |
| *#55* | NBNC | ND / F4 | - / - | - / - | 2 / 2 |
| *#56* | NBNC | G1 / F4 | - / - | - / - | 2 / 0 |
| *#57* | NBNC | G1 / F4 | - / - | - / - | 1 / 2 |
| *#58* | NBNC | ND / F4 | - / - | - / - | 4 / 1 |
| *#59* | NBNC | G2 / F4 | - / - | - / - | 3 / 1 |
| *#60* | NBNC | ND / ND | - / - | - / - | 5 / 1 |
| *#61* | NBNC | ND / ND | - / - | - / - | 5 / 0 |
| #62 | *NBNC* | *ND / ND* | *- / -* | *- / -* | 3 / 1 |
